# Editorial: New developments in mechanical ventilation

**DOI:** 10.3389/fmed.2023.1234419

**Published:** 2023-06-22

**Authors:** Savino Spadaro, Stephen E. Rees, Oriol Roca

**Affiliations:** ^1^Intensive Care Unit, Translational Medicine, University of Ferrara, Ferrara, Italy; ^2^Respiratory and Critical Care Group, Department of Health Science and Technology, Aalborg University, Aalborg, Denmark; ^3^Departament de Medicina, Universitat Autònoma de Barcelona, Bellaterra, Spain

**Keywords:** non-invasive ventilation, acute respiratory failure, ARDS, respiratory monitoring, weaning, electrical activity of the diaphragm, neurally adjusted assist ventilation, weakness assessment

This Research Topic collection entitled “*New developments in mechanical ventilation*”, involving papers with different prospective, confirming that there is a continuous interest in understanding the pathophysiological mechanisms by advanced monitoring useful for preserving the functionality of the respiratory muscles and lungs (1). The effects of hypercapnia in ARDS patients are not completely understood. One of the things that may influence the effect of CO_2_ on the lung is the way how hypercapnia is generated. Spinelli et al. compared the effect of different strategies to generate hypercapnia and their mechanisms of lung protection in an experimental model of unilateral pulmonary artery ligation. Interestingly, full bilateral lung protection (lower histological score, higher regional compliance, lower wet-to-dry ratio, and lower degree of inflammation). In contrast, when hypercapnia was generated by using low tidal volume ventilation or by adding an instrumental dead space, it does not protect the left ligated lung. Of note, inhaled CO_2_ was associated with a lower degree of overdistension in the right lung and increased perfusion of the left lung. This study provides the rationale for testing the effect of CO2 inhalation in patients with ARDS and high dead space fraction to increase lung protection. In this issue, Lescroart et al. analyzed the hemodynamic effects of Time-controlled adaptative ventilation (TCAV) in a swine model of ARDS. One of the main concerns of using TCAV is that it may be associated with a significant hemodynamic impairment due to the high intrathoracic pressures during the prolonged inspiratory phase (CPAP - Phigh). Compared with low tidal volume ventilation, TCAV was not associated with any change in systemic arterial blood pressure, pulmonary blood pressure or cardiac output. Moreover, driving pressure and lung elastance was significantly lower with TCAV, suggesting that TCAV may be potentially useful in ARDS patients (Lescroart et al.). Tailoring protective mechanical ventilation approach based on lung and respiratory muscle physiology is crucial in the future of mechanical ventilation practice. In this issue, Palamim et al. verified the role of comorbidities (such as diabetes mellitus, systemic arterial hypertension, and older age) to determine the outcomes of patients undergone to mechanical ventilation in ICU. Furthermore, they showed that the use of PEEP level >8 cmH2O at admission could be a marker of potential severe hypoxia, associated with increased mortality (Palamim et al.).

Of particular interest, the paper proposed by Cammarota et al. that showed how the patient discomfort during Non-invasive ventilation (NIV) play a role to avoiding intubation and improving survival in patients with acute ARF. Indeed, several aspects should be considered to improve patient adaptation, i.e., the ventilator setting. The use of electrical activity of the diaphragm (EADi)-driven ventilation has been demonstrated to improve patient comfort. Another goal of MV is to guarantee an adequate coordination between the patient's respiratory activity and the assistance provided by the mechanical ventilator. The mismatch between the demand of patient and the level of assistance may produce a patient-ventilator asynchrony and leads to poor clinical outcomes. In this issue, Longhini et al. underline how is crucial to identify promptly the patient–ventilator asynchronies by advanced monitoring or automated software, in order to optimizing the strategies for improving the synchronization of patient-ventilator, using advanced mode of ventilation in adult and pediatric patients. Growing evidences suggest that the use of neurally adjusted ventilatory assist (NAVA) mode, guided by electrical activity of the diaphragm, optimizes patient-ventilator synchronization and avoids both over and under assistance, both conditions that can worsen diaphragmatic function, respectively, causing fatigue or atrophy (2). In a systematic review and meta-analysis conducted by Wu et al., they analyzed the beneficial and physiological effects of NAVA mode in adult patients compared to conventional mode of ventilation, offering a deep analysis of the potential physiologic benefits that may help to identify who can benefit of this strategy. The preservation of diaphragmatic function is a crucial during MV and in particular during the weaning from MV. In elegant pilot study, Bertoni et al. pointed out the role of limb intensive care unit-acquired weakness in ICU and how can play a relevant role in the weaning process. In the last research, Zheng et al. showed that the prophylactic combined use of NIV and high flow nasal cannulae (HFNC) after extubation could be an effective strategy to prevent reintubation in selected patients with high-risk of failure.

In conclusion, this Research Topic pays particular attention to recently progress made on use of innovative mode of ventilation, ventilation strategy and respiratory muscle monitoring, which is expected to provide new insights into research.

## Author contributions

SS, OR, and SR conducted the manuscript. OR and SR the final amendments and approved the final version. All authors contributed to the article and approved the submitted version.
